# Expecting parents’ perceptions of the digital parental support “childbirth journey” constructed as a serious game—an intervention study

**DOI:** 10.1177/20552076221097776

**Published:** 2022-05-16

**Authors:** Caroline Bäckström, Tanja Rolfson, Henrik Engström, Rajna Knez, Margaretha Larsson

**Affiliations:** 1 Research Group Family Centered Health (FamCeH), 101081 School of Health Sciences, University of Skövde, Skövde, Sweden; 2 Regionhälsan Midwifery Unit, Västra Götalandsregionen, Sweden; 3 School of Informatics, University of Skövde, Skövde, Sweden; 4 Skaraborgs Hospital, Skövde, Sweden

**Keywords:** Digital health, general, pregnancy, medicine, Apps, personalized medicine, public health, disease, health informatics

## Abstract

**Objective:**

The aim of this study was to explore expecting parents’ perceptions of the Childbirth Journey as an intervention that includes medical information for parental support, constructed as a serious game.

**Methods:**

In this qualitative study, semi-structured interviews were held with expecting parents in Sweden who were able to talk about specific parts of the Childbirth Journey they appreciated or found difficult to understand. A phenomenographic methodology was employed for data analysis.

**Results:**

Participants perceived the Childbirth Journey to be easily accessible and customized with reliable information. The design and features of the intervention were perceived by the expecting parents to enhance the intervention’s usability, appeal, and trustworthiness. When parental couples used the Childbirth Journey together, it gave them an opportunity to discuss and better understand each other’s situation. The participants proposed several changes to the existing version of the game, mostly related to extending practical information and illustrated scenarios but also to the further development of the game’s design and animations. The participants found the Knowledge portal to be the most appealing part of the Childbirth Journey.

**Conclusions:**

The Childbirth Journey intervention was concluded to be a valuable digital complement to in-person professional support, especially given the current COVID-19 pandemic restrictions in place in Sweden, which do not allow antenatal visits by partners. However, in its current form, the Childbirth Journey has some deficiencies and would therefore benefit from further development and exploration.

## Introduction

Since digital sources are easily available to expecting parents, their access to information is almost unlimited. This capacity to independently search for information can be seen as a benefit.^1^ However, it can also be seen as a challenge for expecting parents, who must assess the trustworthiness of large amounts of information.^2,3^ To accommodate expecting parents’ need for trustworthy digital information, the *Childbirth Journey* was developed as a story-driven serious game by a collaboration of professionals, researchers, and a game company. The *Childbirth Journey* was formulated based on national Swedish and regional regulations and routines as well as current research in the field. The present study focused on exploring expecting parents’ perceptions of the *Childbirth Journey* as an intervention that includes medical information for parental support, constructed as a serious game.

## Background

The rapid digitalization of society is influencing all aspects of human life.^4^ Digital sources have become increasingly important for expecting parents to obtain information related to pregnancy, birth, and child care.^5^ For expecting parents, digital sources are also a crucial means to network with others in the same situation^3,6^ and to share experiences.^6,7^ Previous research has shown that expecting mothers use digital sources to prepare themselves for parenthood and to identify themselves as parents.^2^ Similarly, expecting fathers regularly use the Internet to search for pregnancy-related information,^3^ advice about parenting and about their relationship with their partner, and ways to emotionally adjust during their transition to parenthood.^8^ Online forums do not always meet expecting parents’ needs since lesbian, gay, bisexual, transgender, and queer (LGBTQ) persons sometimes feel excluded from discussions about pregnancy-related issues on such forums.^9^ Expecting mothers require digital technology to assist them in daily life and parental preparations. They turn to digital sources for reassurance that their pregnancy experiences are normal.^10^ Sometimes, expecting mothers self-monitor their pregnancy, which midwives are generally ambivalent toward, according to a systematic review.^11^ Another systematic review that explored expecting parents’ use of digital sources described parents’ need to be guided by healthcare professionals, such as midwives, in selecting relevant and credible information. The review concluded that expecting parents’ health seems to be influenced by their use of digital sources—both positively and negatively—since it can both alleviate and worsen feelings of anxiety. Regardless, their use of digital sources influences their transition to parenthood.^12^

During pregnancy, parents experience a major life transition, one that is decidedly unique.^13^ Becoming a parent has been described as a defining life change for both individual parents and the parental couple.^14^ The process of becoming a family can entail the risk of increased dissatisfaction and tension in the parental couple and can elicit challenging feelings, such as feeling overwhelmed or confused. The transition to parenthood can be hindered by factors such as having unrealistic expectations of parenthood, feeling unprepared for imminent responsibilities, losing control and experiencing tremendous stress. On the other hand, the transition to parenthood can be facilitated more smoothly by viewing it as a natural step in life and by receiving adequate support, both social and professional.^13^ Expecting parents can be strengthened by support that contributes to their understanding and feeling of being prepared for childbirth and parenthood. This support, provided by professionals as well as social contacts, can include practical information about how expecting parents can better prepare for childbirth and parenting. Furthermore, preparation by expecting parents can be bolstered through mutual involvement in each other's role, which can be reassuring for both parents and can reinforce their capacity for mutual support.^15^ Although in Sweden and several other countries, midwives are the primary caregivers responsible for normal pregnancy and birth,^16^ which healthcare professionals are responsible for antenatal care differs internationally. Within antenatal care, midwives, or other healthcare professionals, strive to identify and meet parents’ individual needs for support.^15^ The use of mobile applications within antenatal care has been shown to improve expecting parents’ adherence to antenatal appointments, facilitate their communication with healthcare providers, and foster greater partner support and involvement.^17^ In this regard, more research is needed on digital solutions to facilitate professional support in antenatal care in Sweden.

Previously, nine characteristics of digital media information regarding pregnancy and early motherhood have been described as important for expecting mothers: *customized, detailed, entertaining, immediate, practical, professional, reassuring, regular,* and *unbiased* information.^6^ In addition, serious games, including narrative games, have been described as effective in making learning meaningful.^18^ With these aspects in mind, we developed a serious game that included medical information for parental support.^19^ Serious games were described by Stokes^20^ as games designed to entertain players while simultaneously providing education and training or encouraging behavioral changes. Hence, the serious game developed for this study, the *Childbirth Journey,* was intended to provide expecting parents with an interactive narrative describing normal pregnancy, birth, and labor. This interactive narrative allowed expecting parents to identify with and make choices for avatars that represented a parental couple in the game. This was done to facilitate the expecting parents’ engagement in their knowledge search to prepare for birth and parenting. The goal of developing the *Childbirth Journey* was to create a digital parental support tool for expecting parents that could improve their knowledge about how to prepare for childbirth and parenthood. The intent of the *Childbirth Journey* was not to replace care meetings between parents and healthcare professionals but instead to complement professional support with a digital support alternative for parents. A further description of the *Childbirth Journey* is included in the methods section. However, to broaden knowledge about how serious games, such as the *Childbirth Journey,* may meet expecting parents’ need for information, further research is required. Therefore, the aim of this study was to explore expecting parents’ perceptions of the *Childbirth Journey* as an intervention that includes medical information for parental support, constructed as a serious game.

## Methods

This study was derived from a larger research study that included mixed methods with both inductive and deductive approaches. The current study constituted “*study A*,” as described in the study protocol by Bäckström et al.,^19^ and focused on an intervention, the *Childbirth Journey,* for expecting parents. For this study, an explorative design, qualitative methods, and an inductive approach were used, and the data were collected through interviews with expecting parents who had received the intervention described below. For data analysis, phenomenographic analysis was used. Originally, phenomenography was developed within the pedagogical traditions; in recent years, however, it has become a well-established approach within nursing research as well. Centrally, within phenomenography, the goal is to describe the various ways in which people understand a phenomenon, and it is the second-order perspective that is explored: “how things are perceived or understood.” In contrast, other qualitative methods aim to describe the first-order perspective instead: “how things really are.”^21^

### Intervention

The intervention includes digital parental support developed as a serious game, the *Childbirth Journey,* for expecting parents in Sweden. Before developing the *Childbirth Journey*, interviews with expecting and new parents were conducted to explore their perceptions of future digital parental support. The results of the interviews identified various features that should be included in future digital parental support tools, such as a greater user-friendliness, more variety and enhanced support for additional languages. The parents considered it important for future digital parental support to provide individualized information and links to external websites of interest for parents. In addition, to make information more engaging, future digital support constructed as a serious game was suggested.^22^ Further, a systematic review was carried out to explore expecting parents’ use of digital sources and how it influences their health during pregnancy. The review showed that digitalized society involves both opportunities and challenges and that expecting parents have a need for a variety of digital sources to improve their health.^12^ These aspects were considered when constructing the *Childbirth Journey* for the current study; however, parents’ request for future digital parental support to include virtual meetings with healthcare professionals^22^ was not prioritized due to financial limitations. The *Childbirth Journey* was developed and implemented collaboratively by healthcare professionals and researchers in antenatal, labor, postnatal and child healthcare, a researcher specializing in serious games, and a game development company. All information included in the *Childbirth Journey* was controlled by the healthcare professionals in the research group. The goal of developing the *Childbirth Journey* was to create a digital parental support tool for expecting parents that could enhance their knowledge about how to prepare for childbirth and parenthood. The *Childbirth Journey* was developed in both Swedish and English so that both Swedish- and English-speaking parents could use it. The *Childbirth Journey* is available as a mobile game and as a stand-alone PC application. In the present study, only the mobile game version was explored. The *Childbirth Journey* constitutes two different parts: (a) a story-driven game and (b) a *Knowledge portal*, described below.

#### The story-driven game

The main reward of a story-driven serious game is immersion in a narrative experience. Narrative has previously been described as effective in making learning meaningful, especially when it includes integrated fantasies, characters, adaptiveness, or responsivity.^18^ In the *Childbirth Journey*, the narrative consists of an expecting parental couple experiencing preparation for birth, the onset of labor contractions, and birth at a labor ward in a Swedish hospital environment. The narrative is divided into three different scenes: (a) home, preparation; (b) home, labor starts; and (c) labor, at hospital. However, four more scenes are planned for development and inclusion in a future version of the *Childbirth Journey*: (d) midwife antenatal clinic; (e) postnatal ward at hospital; (f) home after childbirth; and (g) midwife postnatal clinic. This study could, therefore, be described as a pilot study, allowing the exploration of the first three scenes before the development of the remaining scenes.

The *Childbirth Journey* was built as a 3D game, as recommended by the developer. The player (i.e. the expecting parent) is able to choose a role as either the pregnant woman or her partner, which means that he/she may choose an avatar that illustrates one's own or one's partner's role. The player can influence the scenarios in the *Childbirth Journey* by choosing among the various options presented. The events in the scenarios unfold differently depending on the options chosen. The different scenarios encompass issues such as the expectant parents’ preparation for childbirth, the start of labor, labor, pain relief, breastfeeding, formula, parental leave and parenthood, among other issues. The *Childbirth Journey* was developed as a story-driven serious game with opportunities to permit expecting parents to influence the different scenarios in the game and thereby obtain and process information based on their individual needs. With this, it was assumed that the parents’ interest in accessing information, as a self-preparation for childbirth and parenthood, would be influenced. The *Childbirth Journey* does not pose any real obstacles to player progression, which means that each scenario starts and ends with normal labor and neonatal outcomes, regardless of which options the parents choose. In this way, the *Childbirth Journey* can be said to belong to the second range grouping in the serious game continuum, proposed by Marsh,^23^^,p.64^ “serious games with reduced gaming characteristics.”

**Figure 1.**
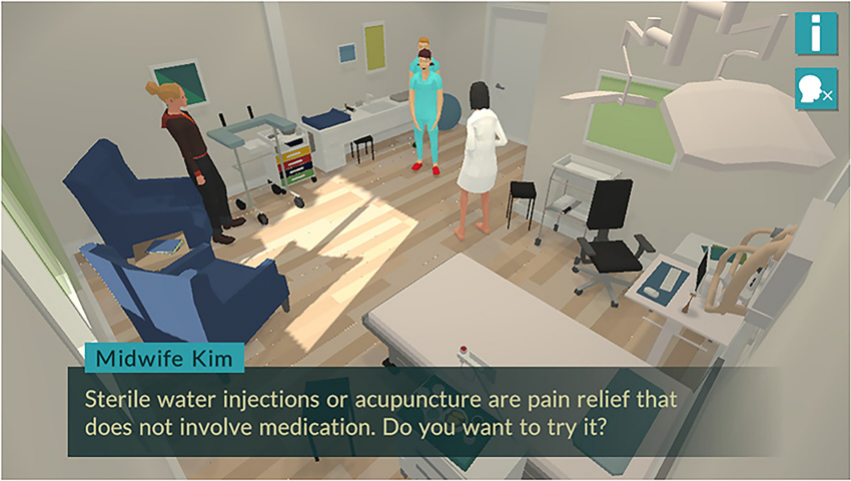
Screenshot illustrating a question asked by the avatar *midwife Kim* in the story-driven game *Childbirth Journey*.

The scenarios presented and the information included in the *Childbirth Journey* are based on evidence, professional knowledge, and routines applied at antenatal units as well as at labor wards in the hospital setting. Through the scenarios in the game, expecting parents are provided with information that depends on the options they choose. For example, in the labor scenario, the parents may choose whether the birthing woman should experience labor pains as manageable or not. Depending on the parents’ choice, the birthing woman avatar in the scenario expresses whether or not she is able to manage the actual situation. Based on this, the healthcare professional avatars (i.e. the midwife or nurse assistant) respond differently to the birthing woman and partner avatars in the game. Also, the expecting parents can decide whether or not the birthing woman avatar needs pharmacological pain relief. Based on this decision, the expecting parents are presented with various options for non-pharmacological and pharmacological pain relief. In Figure 1, for example, the avatar *Midwife Kim* answers the birthing woman's question about which types of non-pharmacological pain relief are available: *“Sterile water injections or acupuncture are pain relief that does not involve medication. Do you want to try it?”*

**Figure 2.**
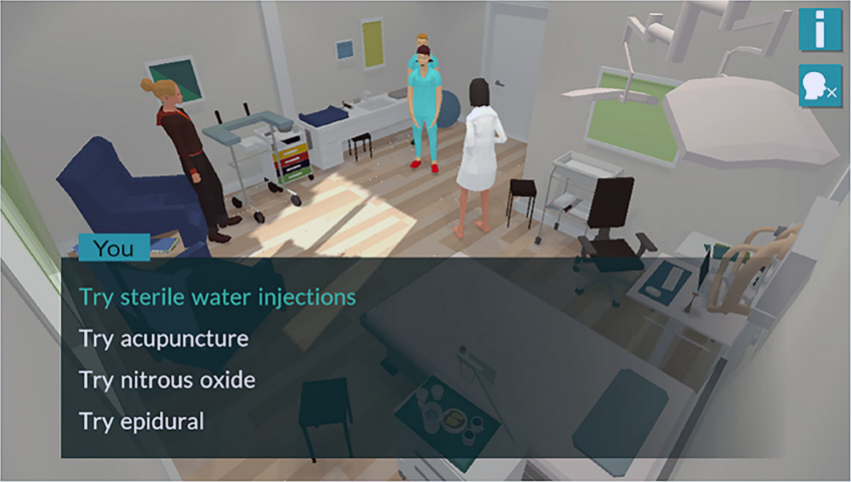
Screenshot illustrating different alternatives posed to the player in the story-driven game *Childbirth Journey*.

If the player answers *“I don't know,”* she/he is given several alternatives: *“Try sterile water injections,” “Try acupuncture,” “Try nitrous oxide”* or *“Try epidural,”* as shown in Figure 2.

In addition to the story-driven game, various films were created that illustrate various conversations between a midwife and a parental couple (film duration between 5 and 15 min). The films also illustrate different examinations that midwives apply during normal labor. For example, one film illustrates a CardioTocoGraphy examination, which is available to the player in the part of the labor scene that illustrates the expecting parents’ arrival to the labor ward at the hospital. Films were also created in which an obstetrician talks about caesarean birth and vacuum extraction. All films were recorded at the labor ward at the hospital that served as the research setting, and the healthcare professionals taking part in the films were all working at the actual labor ward. All films are also available through the *Knowledge portal,* as described below. In the story-driven game, different web links appear depending on the choices the player makes, each of which provides the user with access to external websites with additional information.

#### The Knowledge portal

**Figure 3.**
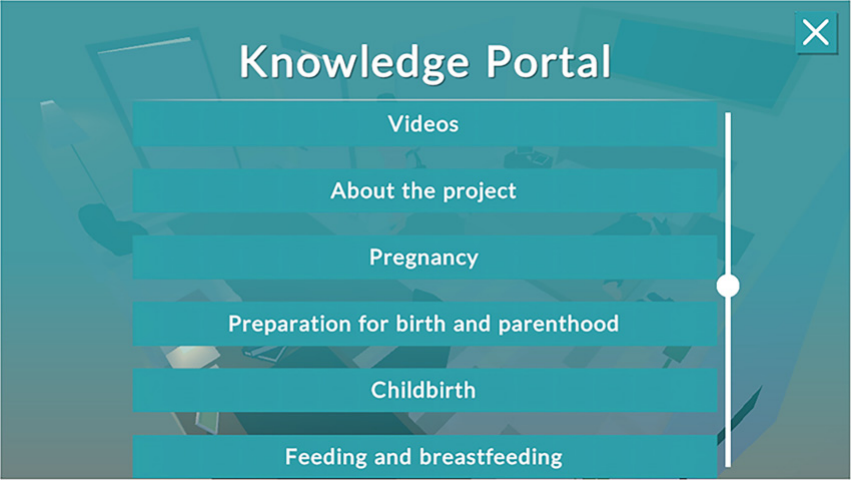
Screenshot illustrating examples of issues in the *knowledge portal* in the *Childbirth Journey*.

The *Knowledge portal* functions as a type of library that includes web links to external websites of interest for expecting parents (such as a Swedish national website that includes health information, www.1177.se, or a website that features Swedish social insurance issues, www.forsakringskassan.se). The *Knowledge portal* also includes web links to podcasts and suggestions for literature in book or article formats. The information included deals with pregnancy, labor, pain relief, breathing techniques, the female body during and after pregnancy, breastfeeding and formula, baby care, the parental role, parental leave, and the parental couple relationship. Also, the films included in the story-driven game are available through the *Knowledge portal*. The *Knowledge portal* is not accessible outside of the *Childbirth Journey*. The player reaches the *Knowledge portal* by clicking on a button marked with an “i,” as shown in Figures 1–2. In Figure 3, some of the issues included in the *Knowledge portal* are shown.

In the current study, parents who agreed to participate were provided with the intervention (i.e. access to download the *Childbirth Journey*). They were instructed to use the *Childbirth Journey* according to their own needs and interests, which meant that they could use it individually or simultaneously with their partner and test the Swedish and/or English versions and the birthing woman and/or partner interface. However, they were instructed to test all three scenarios in the serious game as well as to visit the *Knowledge portal*.

### Settings and participants

The current study was conducted within a setting representative of the Swedish population, including both rural and suburban residents. The setting included approximately 280,000 inhabitants and one hospital with a labor ward. Participants were recruited using convenience sampling. Social media was used to advertise the opportunity for parents to participate in the intervention. Besides, midwives at antenatal units within the setting asked expecting parents whether they would be interested in participating during a gestational assessment in the third trimester. In total, seven expecting parents gave their consent to participate, all of whom contacted the research project leader (CB) for further information about the study. All of the expecting parents who gave their consent to participate received the intervention and were included in the study. The seven expecting parent participants (four expecting mothers and three fathers) were all between 29 and 36 years old, were all born in Sweden, and were all university graduates. Four of the participants were expecting their first child, while the remaining three participants had a previous child. All of the participants planned to give birth at the hospital chosen as the research setting, which meant that they were cared for at the setting's antenatal units and had knowledge of local routines. Therefore, they were considered suitable to take part in the intervention, as the *Childbirth Journey* included information about local routines. Among the seven participants, there were three parental couples and one expecting mother who participated without her partner.

### Data collection

For this study, data were collected through semi-structured interviews. Before the interviews, an interview guide was developed that included open-ended and follow-up questions, such as *Please tell me about your perceptions of the Childbirth Journey; Which parts of the Childbirth Journey did you appreciate more than others?; Were any parts of the Childbirth Journey difficult to understand?;* and *What has your usage of the Childbirth Journey meant for you?* Before conducting the interviews, the interview guide and technique were tested with two individuals. The results of the interviews showed that the interview questions were easy for the interviewees to respond to, and the technique was considered functional. The interviews were not included in the data analysis. All interviews were held digitally using a video link so that the interviewees and the interviewer were able to see each other. The reason for conducting the interviews digitally instead of face to face was the current COVID-19 pandemic, which led to restrictions on opportunities for participants and researchers to meet in real life. Three of the interviews were held with the parental couple together, according to the participants’ request, and one interview was held with an expecting mother individually (i.e. the participant's partner did not participate in the study). In total, seven parents were interviewed. The interviews were held approximately 2 weeks after the participants had received the intervention by two researchers (CB and ML) with doctoral degrees and previous experience conducting qualitative interviews. The interviews lasted between 35 and 55 min.

### Data analysis

**Figure 4.**
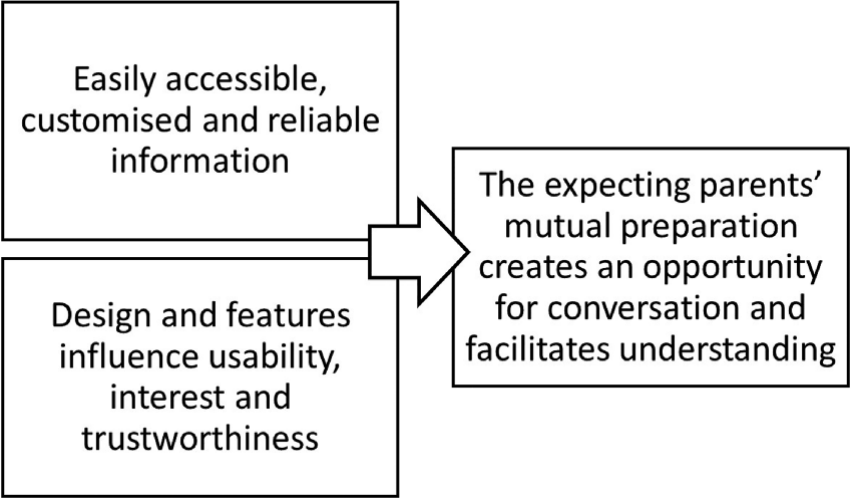
The findings in relation to the “outcome space” and the hierarchical arrangement of the descriptive categories.

For data analysis, the seven steps described by Sjöström and Dahlberg^24^ were used. Initially, in the first step, the transcripts (46 A4 pages, 1.0-spaced type) were repeatedly read and discussed by two of the authors (CB and TR) to gain a holistic understanding of the content (familiarization). Then, in the second step, the most significant parts, that is, those that specifically addressed the aim of the study, were identified (compilation). Thereafter, comparisons were made to identify similarities and differences (i.e. third step: condensation), which were grouped and interrelated (i.e. fourth step: grouping). Further comparisons of similarities and differences were made to distinguish between the groups, which was the fifth step (comparison). In this step, four of the authors participated in mutual reflective communications (CB, TR, RK, and ML). From this, three descriptive categories arose, which were named (i.e. sixth step: naming) and then compared to identify logical relationships between the descriptive categories (i.e. seventh step: contrastive comparison). The relationships between the descriptive categories were illustrated in the outcome space presented in Figure 4. The authors had various levels of expertise with qualitative methods and phenomenographic analysis, yet they all had a mutual understanding of interview analysis.

### Ethical considerations

The study was approved by the Regional Ethical Review Board in Gothenburg Sweden (Dnr: 2020-01689). The intervention was considered low risk; however, some negative experiences may be relevant for the participants, such as experiences related to using the *Childbirth Journey* or problems with technology. Such experiences may lead to disappointment or frustration among the participants, with possible dropouts. On the other hand, the intervention may lead to positive effects for the expecting parents, such as increased knowledge and feelings of being better prepared for the upcoming birth and parental role. The participants received study information in both written and verbal form before giving consent to participate. They were able to choose a time for the interview themselves, and the interview recordings were stored securely, accessible only to the researchers. Participants’ identities were kept confidential. Quotes were translated from Swedish to English by two of the authors (CB and TR). The quotes are presented in the results section without revealing the participants’ identities.

## Results

The data analysis resulted in three descriptive categories and corresponding perceptions as presented in Table 1.

**Table 1.** Descriptive categories and perceptions.

Descriptive categories	Perceptions
Easily accessible, customized and reliable information	Easily accessible and customized information that meets expecting parents’ needs Reliable information provided by professionals Information provided in various ways facilitates expecting parents’ understanding
Design and features influence usability, interest and trustworthiness	Authentic sound recordings are more trustworthy than synthetic speech Graphics and animations influence usability, interest, and trustworthiness Opportunity to navigate, pause and return influence usability
The expecting parents’ mutual preparation creates an opportunity for conversation and facilitates understanding	Usage of the *Childbirth Journey* creates an opportunity for conversations between the expecting parents Using the Childbirth Journey facilitates understanding of each other's situation Using the Childbirth Journey creates the opportunity for partners to mutually prepare despite COVID-19 restrictions

### Easily accessible, customized, and reliable information

This descriptive category included the expecting parents’ perception that the overall information provided in the *Childbirth Journey* was easily accessible. The expecting parents could return to the information repeatedly, which made it easier for them to obtain it. The information was perceived as customized to meet the expecting parents’ needs. The information was described as reliable because it was developed and controlled by healthcare professionals and researchers. The expecting parents perceived that their knowledge and understanding were facilitated by the various ways in which the information was provided.

#### Easily accessible and customized information that meets expecting parents’ needs

The expecting parents perceived the information provided in the *Childbirth Journey* to be easily accessible, customized and relevant to their needs, which they considered satisfactory. They were positive about the information that was delimited compared to other information for which they searched on the Internet. The application format, including web links to external websites, was perceived positively. One participant compared the web links included in the *Childbirth Journey* with the web links that were distributed on paper by the midwife at the antenatal unit and explained that the paper web links must be manually typed into one's own browser, which could cause frustration. The web links provided in the *Childbirth Journey* were, in contrast, clickable, leading directly to the website. Such easy access to various information made it possible for the expecting parents to decide for themselves at what pace they could obtain the information. This gave them time to process, evaluate, and reflect on the information. It also enabled them to return to the information and access it repeatedly. These perceptions were more common regarding the information provided in the *Knowledge portal* than the information in the story-driven game.

The expecting parents wanted more scenarios in the story-driven game that would illustrate complicated childbirth, postnatal care at the hospital, and the first days at home with their child. Some mentioned that they would have appreciated access to the scenes not yet developed as well. They wanted more practical information about how they could prepare their home for their child's arrival (i.e. which type of diapers, clothes, or furniture were needed) and what they needed to pack in their bag before their hospital stay (such as diapers and pads for the mother): *I would like to have more information about what to bring* [to the hospital], *apart from the car seat and toothbrush. Yes in some way you understand that, but just other pieces. Do I need to bring diapers and clothes and stuff like that?* (Informant 4).

According to the expecting parents, their use of the *Childbirth Journey* increased their understanding of issues relevant to pregnancy, childbirth, and parenthood. This improved their understanding of what questions to ask in care meetings with midwives.

#### Reliable information provided by professionals

A common perception was that it was beneficial that the *Childbirth Journey* was developed and controlled in collaboration between researchers and healthcare professionals in clinical work. The expecting parents explained that this made the information more reliable, which, in turn, strengthened their feelings of trust towards the *Childbirth Journey* overall. For example, the midwife in the films was calm, which made her trustworthy; the films visualized the environment at the labor ward at the hospital, which helped the expecting parents to better understand what would happen during the upcoming birth. It also bolstered their ability to understand what kind of cognition (such as individual mental goal targets, for example) they could use to manage birth and labor contractions.

Further, the external information sources included in the *Knowledge portal* (i.e. literature references, external web sites, etc.) were perceived to be reliable because the participants understood them to be grounded in research, evidence, professional knowledge, or local routines at the hospital in the research setting: *In other words, reliable information, produced by those who are well read and have good knowledge of the issue, who are legitimized. That the information is reviewed and controlled by experts and not based on one's own opinions* (Informant 5).

The parents perceived the *Childbirth Journey* as time saving because it gave them access to information that, in turn, reduced their need to contact the midwife about issues that could easily be answered via the *Childbirth Journey* instead.

#### Information provided in various ways facilitates expecting parents’ understanding

The expecting parents appreciated the various ways in which information was provided in the *Childbirth Journey*, that is, different scenarios in the story-driven game, films, and the Knowledge portal. Some of the expecting parents perceived the normal birth scenario presented in the story-driven game as reassuring and satisfactory, while others asked for more scenarios describing complicated birth scenarios (i.e. caesarean section and post-partum hemorrhage). One expecting mother, who had given birth previously, perceived that the birth scenario presented in the story-driven game reassured her about her previous birth experience: *I think it was because you recognize yourself in the situation* [the birth scenario in the serious game]. *It was reliable. Then, I thought that the midwife in the film felt like she had done this many times* (Informant 2). The opportunity to choose what would happen in the birth scenario was appreciated because the expecting parents could obtain information based on their individual needs. This meant that the information became more relatable, contributing to their feeling of having control, being secure and being mentally prepared for childbirth and parenthood.

*To be able to go through the whole process at your own pace and decide for yourself. For example, I chose the outcome that the child does not breathe so well to see how such a scenario could unfold. It was probably also a good thing to experience, so that if it happened, I would know how and why it happened. It felt good. It is, as always, something you worry about, the child being sick or injured in some way. So it was something new. I had never seen that before. Then, I also took the option that a piece of the placenta was left* [in the uterus] *and that they then needed to call for an extra midwife and physician. So, it felt good to be able to see alternatives that are possible, which I myself had not experienced previously. It provided me with a feeling of strong mental preparedness. So, it was something I thought was very good, to be able to see things that can happen.* (Informant 1)

On the other hand, some expecting parents felt that choosing among multiple options in the story-driven game slowed the process of obtaining information. They would, instead, have appreciated having the information provided in films structured as traditional constructive and information-rich lectures. These expecting parents also noted having had no previous personal interest in playing serious games.

The expecting parents perceived the information provided in the films to be easy to obtain and understand, especially when they experienced fatigue. This is because, when fatigued, it can be difficult to read text and comprehend its content. As such, they perceived the opportunity to watch and listen to films instead of reading text to impose less strain. A common perception was that the user experience of the *Childbirth Journey* could benefit from the inclusion of more films illustrating which breathing techniques to use during birth, which massage techniques the partner could use to support the birthing woman, types of pain relief other than nitrous oxide (which was already present in a current film), complicated birth, breastfeeding, and the first time at home with the baby. One expecting mother expressed that it could be valuable to have various midwives, or other healthcare professionals, take part in the films, since doing so could increase familiarity with the midwives and healthcare professionals when meeting them in real life while arriving at the labor ward. In turn, this could stimulate a feeling of calmness and reduce anxiety, according to the expecting mother: *Then, I really thought these films were very good. You could have even more* [films], *I think. I thought it was good when they explained a bit like this: “I will call* [for an anesthesiologist], *because you have said that you want an epidural… then it goes like this.”* *…* *That they asked, it is also very good I think. How you feel about this, and how you feel about that. Then you know a little about what to expect.* (Informant 2)

### Design and features influence usability, interest, and trustworthiness

This descriptive category describes the expecting parents’ perception that the design and features of the *Childbirth Journey* influenced both its usability and appeal, making the expecting parents more interested in and trusting when using it. More specifically, they perceived the authentic sound recordings to be more trustworthy than the synthetic speech in the story-driven game. They also expressed the need for further development of the design, animations, and navigation possibilities of the game.

#### Authentic sound recordings are more trustworthy than synthetic speech

The expecting parents highlighted several deficiencies in the story-driven game that influenced its usability and their interest in using it, such as incorrect pronunciations in the synthetic speech, which could lead to an occasional loss of focus on the game and its content. In fact, instead of promoting serious involvement in the game, such inaccuracies in synthetic speech often sparked amusement: *At some point we laughed at how the computer voice expressed how to breathe. When a computer reads a text, sometimes there are some funny emphases. Yes, at some point we focused on that instead. That was a little bit fun* (Informant 6).

In contrast, the recorded human voice was perceived to be more trustworthy, and consequently the expecting parents requested that the synthetic speech be replaced with real recordings. Moreover, a human narrator's voice can emphasize different expressions, such as breathing techniques, which cannot be emphasized by the current synthetic speech. Besides, the expecting parents asked for the inclusion of more diegetic sounds in the story-driven game, such as pouring water when the shower was used, playing music when the avatars turned on a radio, or playing screams, wails or other noises made by babies when they are born.

#### Graphics and animations influence usability, interest, and trustworthiness

According to the expecting parents’ perceptions, the graphics in the story-driven game could be improved, as they seemed to be outdated. Additionally, the avatars’ positions and postures were sometimes inauthentic or unbelievable, such as when the healthcare professional avatars stood far away from the birthing woman avatar at the same time as they were said to be examining her. Altogether, this influenced the expecting parents’ interest in using the story-driven game and the perceived trustworthiness of the game overall.

All expecting parents felt that the story-driven game was not the most appealing part of the *Childbirth Journey*. Instead, they expressed greater appreciation for the films and the *Knowledge portal*. This is primarily because of the technical errors in the game, such as the incorrect positioning or placement of the avatars. Consequently, the expecting parents believed that the animations needed improvement to become more trustworthy: *Yes, we concluded that the game itself was not really something that suited us. But we thought that the films were good. We haven't watched all the films that were available, but we perceived them as more useful than the actual game dialogues* [between the avatars] *that came up…* *The voice was much computerized… It became very monotonous. The films were a little more lively and easier to absorb in some way* (Informant 6).

#### Opportunity to navigate, pause, and return influence usability

The opportunity for the expecting parents to navigate between different parts of the *Childbirth Journey* (i.e. the story-driven game, the *Knowledge portal,* and external websites) was perceived to be entertaining. This was important for the game experience and for the expecting parents’ interest in playing. However, the game lacked functions that would allow the expecting parents to pause the films or return to previous scenarios in the story-driven game: *It might have been good to have a return function where you can go back. Because, at one point, I accidentally pressed a button and got forwarded in the text. Then, it would have been quite nice that, instead of jumping back to the beginning, you should be able to go back* [only] *one step to be able to read* [the text] *again* (Informant 5).

Furthermore, the expecting parents wanted clickable links in the story-driven game. Currently, the *Knowledge portal* must be accessed in order to click on links to external websites or films, which could cause frustration. The expecting parents perceived the story-driven game to be slow, and therefore not very appealing, and as such commented that the game should be sped up. Overall, the expecting parents perceived that the story-driven game could be improved to increase its usability and the general game experience. However, one expecting parent remarked that he was not interested in using the story-driven game again, even though it would be improved, due to his general lack of interest in digital games.

### The expecting parents’ mutual preparation creates an opportunity for conversation and facilitates understanding

The expecting parents used the *Childbirth Journey* in various ways. Some parental couples used it simultaneously, while others used it individually. Nevertheless, a common perception was that using the game stimulated conversations between the parental couple, which in turn facilitated mutual understanding of each other's situation.

#### Using the Childbirth Journey creates an opportunity for conversations between the expecting parents

The expecting parents used the *Childbirth Journey* in various ways: Some parental couples used it simultaneously, which meant that they used the same device. This stimulated conversations between the expecting parents about the content or design of the *Childbirth Journey* as well as their own preparations for childbirth and parenthood. Some of the expecting parents explained that they used the *Childbirth Journey* individually and afterwards communicated with their partner about its content and the knowledge they obtained from it. These expecting parents perceived the *Childbirth Journey* to be a platform helpful in mutually preparing them for childbirth and parenthood, which could in turn strengthen and deepen their relationship. This is because the issues raised in the *Childbirth Journey* enhanced the expecting parents’ understanding about issues of importance to them and how to communicate these issues with each other. Such improved communication could assist the parental couple in avoiding unnecessary conflicts because it clarifies their wishes and expectations prior to becoming parents (i.e. parenting, parental leave, and breastfeeding and/or formula).

That was probably a reminder for us to discuss the parental role. *What views we have, that it becomes clear what is important to me and to my partner. Because after a while you notice that you* [the parental couple] *have different opinions about things. I think it is very good for us to have talked about it before* [the arrival of the baby], *to avoid unnecessary conflicts, especially if it is sensitive issues that are being addressed, like how to distribute parental leave*. (Informant 1)

#### Using the Childbirth Journey facilitates understanding of each other's situation

That the story-driven game had one interface for the pregnant woman and another for the partner was positively perceived, as this framework created an opportunity for the expecting parents to gain insights into each other's roles. This in turn facilitated their understanding of each other's particular situation. Some expecting parents perceived that using the *Childbirth Journey* had beneficially affected their relationship, permitting them to share their experiences and promoting mutual preparation for parenthood. Others, however, did not believe that the game had any impact on their relationship. The parental couples who were interviewed together had the same perceptions concerning whether the *Childbirth Journey* had influenced their relationship.

*I think it was very good that you could let your partner* [the avatar shaping the partner in the story-driven game] *ask questions, and that you could ask questions yourself, leading to the reflection that we actually are in this together. I thought it was very good. You were able to choose a little like this from time to time. I think it's useful to reflect on the other′s role. You are not there yourself, so to speak*. (Informant 2)

#### Using the Childbirth Journey creates the opportunity for partners to mutually prepare despite COVID-19 restrictions

The partners (i.e. expecting fathers) talked about the current COVID-19 pandemic and the implications of its associated restrictions for them. For instance, the restrictions prevented them from attending prenatal check-ups with the midwife at the antenatal unit and restricted their access to parental groups. Thereby, they lacked exposure to and interactions with other expecting parents (i.e. parental groups) as arranged by midwives. In this regard, digital parental support, such as the *Childbirth Journey*, could be important for expecting parents to mitigate such restrictions, thereby functioning as a complement to parental group meetings in real life. The parental couple's simultaneous access to professional parental preparatory support could be facilitated by the *Childbirth Journey*: *It might become easier to remember issues, if you use some kind of game from which you have discussed things, to remember and explain it in an easier way. Yes, especially during these times* [COVID-19 pandemic]*, as I said previously, I am not allowed to participate there* [at the antenatal unit] (Informant 5).

### The outcome space

The phenomenographic method used for data analysis in the current study permitted the examination of logical relationships between the descriptive categories. More precisely, the ways in which expecting parents perceived and reflected on their experiences from using the intervention, *Childbirth Journey,* were analyzed. A hierarchical arrangement of the descriptive categories arose during the analysis in which the expecting parents’ understanding of their use of the *Childbirth Journey* was considered. The relationships between the descriptive categories, as well as how they comprise a whole, are illustrated as an outcome space in Figure 4. In the outcome space, the descriptive categories were arranged based on theoretical assumptions regarding frequency (i.e. how often the descriptive category was mentioned by the expecting parents), position (i.e. how early in the interviews the descriptive category was mentioned by the expecting parents), and pregnancy (i.e. how significant the descriptive category was to the expecting parents).

The descriptive categories *easily accessible, customized and reliable information* and *design and features influence usability, interest and trustworthiness* were the most prominent in the interview narratives; they were often mentioned early on by the expecting parents during the interviews (position), and they were often repeatedly mentioned (frequency). Therefore, these two descriptive categories were placed to the left (illustrating the beginning of the process) in the outcome space (Figure 4). The expecting parents’ perceptions of the information included in the *Childbirth Journey (easily accessible, customized and reliable information)* and its design *(design and features influence usability, interest and trustworthiness)* facilitated conversations between the expecting parents and enhanced their understanding of each other's situation. From a theoretical perspective, the expecting parents’ perceptions of how the *Childbirth Journey* intervention facilitated their conversations and understanding were the most significant for them (pregnancy). Therefore, the descriptive category *the expecting parents’ mutual preparation creates an opportunity for conversation and facilitates understanding* was placed to the right (illustrating the end of the process) in the outcome space (Figure 4), with an arrow (i.e. from the left to the right) highlighting how the categories relate to one another, from a theoretical perspective.

## Discussion

The results of the current study showed that the *Childbirth Journey* intervention, as a digital parental support tool developed as a serious game, was perceived by the participants to contain easily accessible, customized and reliable information. This information was perceived to be reliable because it was controlled by professionals and researchers. In addition, the information was perceived to meet the expecting parents’ general needs for information. Previously, nine characteristics of digital media information regarding pregnancy and early motherhood have been described as being the most important for expecting mothers: *customized, detailed, entertaining, immediate, practical, professional, reassuring, regular,* and *unbiased* information.^6^ From this, we can conclude that the *Childbirth Journey* intervention meets the need for *immediate, customized,* and *unbiased* information controlled by *professionals*. Becoming a parent might be an overwhelming experience in terms of the sheer volume of information required by the expecting parents. Today, it is common for expecting parents to have access to the Internet, which they often rely on to retrieve information about pregnancy, childbirth, and the expected child.^3,5,6,10,25–27^ This practice could be described as self-education.^2^ However, Swedish research has shown that more than one-half of expecting mothers and fathers (65.6% and 61.8%, respectively) are at some point concerned about the accuracy and reliability of the pregnancy-related information they read on the Internet.^3,27^ Consequently, fulfilling expecting parents’ need for accurate and reliable information could enhance their understanding about how to prepare for childbirth and parenthood. Such information could be provided by both professional support (from midwives or other healthcare professionals) and social support (from family, friends or significant others) that is grounded in expecting parents’ individual needs.^15,28^ Hence, we developed the intervention, *Childbirth Journey,* with the goal of providing expecting parents with a digital support tool that could expand and improve their knowledge concerning childbirth and parenthood.

The results of this study showed that the various ways in which information is obtained through the *Childbirth Journey* were perceived to enhance expecting parents’ knowledge and understanding. This is because the tool provided the expecting parents with opportunities to decide for themselves at what pace they could obtain, process, evaluate, and think about information. Besides, as the intervention can reassure expecting parents about the reliability and accuracy of the previous knowledge and birth experiences they have obtained, it also demonstrates the digital media information characteristic *reassuring,* which has been described as valuable by Lupton.^6^ This result is compelling since expecting parents desire knowledge and understanding with respect to how they can better prepare for childbirth and parenthood,^13^ which can in turn more smoothly facilitate their transition to parenthood^13,15^ and promote feelings of calmness, comfort, and reassurance.^6,10^ However, expecting fathers and co-mothers (i.e. lesbian mothers) may feel excluded from professional support during pregnancy, which may adversely affect their transition to parenthood.^13,29^ Moreover, the feeling among expecting fathers of being unprepared to support the mother during birth may culminate in fears of childbirth.^30^ Whether or not the intervention in this study attenuated expecting parents’ fears of childbirth were not revealed in the results even though the participants expressed that their sense of being prepared for childbirth had been improved. The lack of a result in this regard might be because the study participants expressed generally normal feelings towards birth, which could be inferred to mean that they had no such fears of childbirth. Therefore, further exploration of the intervention specifically with respect to fear of childbirth is needed. The idea for developing the *Childbirth Journey*, as a tool to provide expecting parents with medical information in the framework of serious game, arose before the COVID-19 pandemic. Since then, however, the need for *digital* education sources has greatly expanded due to pandemic restrictions and their general implications and ramifications.^31^ This was evident in the perceptions of the expecting parents interviewed in our study, who emphasized the more significant role of digital sources, like the *Childbirth Journey,* for parental support and preparations for childbirth and parenthood. This is in line with previous research that has shown that expecting mothers turn to digital sources when they feel abandoned by medical professionals^32^ in order to obtain additional information, leading to enhanced feelings of control.^33^ However, further exploration of alternative digital support tools for expecting parents as a complement to regular professional support is needed.

The results of this study showed that the participants used the *Childbirth Journey* intervention in various ways, with some using it simultaneously and others individually. Regardless, the intervention facilitated conversations among the expecting parents about issues they believed were important to discuss. The participants also perceived the opportunity to choose an interface in the story-driven game as beneficial since it allowed them to gain insights into each other's roles—indeed, this was one of the intentions of developing two interfaces. More specifically, two interfaces were developed so that both parental roles could be clarified and better understood, especially since the role of the partner (the parent who is not pregnant) is sometimes undervalued, neglected, or even excluded in care meetings with healthcare professionals.^15,34–36^

Since the development of the *Childbirth Journey* was initiated and supervised by healthcare professionals, this digital tool can be considered a valid form of professional support. Such support has previously been shown to facilitate communication and understanding among parental couples. In turn, improved communication skills between expecting parents promote feelings of togetherness^15^ and strengthen the co-parenting relationship, which is defined as the ways in which parents work together in their roles as parents, each of which are invaluable for parenting and parental adjustment, as well as for child outcomes.^37,38^ Furthermore, professional support has been shown to improve the quality of the relationship between expecting parents and to lower the risk of separation.^15^ However, none of the participants in this study perceived that the *Childbirth Journey* intervention had affected their relationship other than encouraging conversations. Nevertheless, improved communication skills by both members of the parental couple can strengthen their ability to work together as a team, which is especially valuable for children since their emotional and cognitive development is dependent on the quality of the relationship between their parents.^39^ Concerning the current study, it could be argued that the expecting parents simply had not had sufficient time to notice whether any effect had occurred on their relationship, as they only had access to the intervention for 2 weeks before the interviews. It might be the case that effects on the relationship occur over longer time periods, which is why further exploration of the intervention from a longitudinal perspective is needed.

Additionally, the results of this study suggest the need for improving the *Childbirth Journey* intervention. Expecting parents proposed several improvements related to the design and features of the story-driven game since these aspects affected its usability and the overall game experience, as well as the participants’ interest in using it and the degree to which they found it to be trustworthy (see Supplemental material, Table 2). The participants were more satisfied with the *Knowledge portal* and the information included in the *Childbirth Journey* overall. The initial ambition of creating a story-driven game was to promote the *entertaining* characteristics discussed above. The initial project team did not possess any expertise in games or game development. Thus, the approach taken was to involve a game development studio that was contracted to develop the game in collaboration with healthcare professionals and researchers in antenatal, labor, postnatal, and child healthcare. A researcher specializing in serious games joined the project team when the development started. The development process suffered from many of the challenges identified in prior research on serious games: the budget for development was limited, which had a crucial impact on design decisions;^40^ there was a gap in perspectives and focus between the project group and the game developers;^41^ the focus on visual and physical fidelity in the simulated and animated elements was emphasized by professionals, while the entertainment aspects were not stressed by the contractor;^42^ the target group, expecting parents, was broad with respect to gaming experience and gaming preference, and it was difficult to make a game that fit everyone since players’ motivations differ.^43^ Besides, the experience and skills of players can affect how they perceive a game; when a system is presented as a game, user expectations tend to be high, and the system is often compared with entertainment games that have budgets that are several orders of magnitude larger than those of typical serious games.^44^ However, the participants’ expectations for the present intervention, *Childbirth Journey,* were not explored.

The combination of factors mentioned above can explain the feedback provided concerning the game elements obtained in the present study. The participants noted that the story-driven game had outdated graphics, animations, and audio that disturbed the game experience. The only game-related element that was considered positive was the option of playing in one's partner's role and interactively asking questions during the game. In retrospect, many of the negative perceptions reported by participants were related to an early design choice to make a 3D game in which the player could control an avatar that navigated the virtual environment. The 3D models and animations required substantial development resources but did not contribute strongly to the core focus of the game, which was to provide a narrative experience. The synthetic speech used for the dialogue in the story-driven game was selected because it was available for free, but it had a strongly negative effect on participants’ perceptions of the narrative. One conclusion that can be drawn from this study is that a much stronger focus on the core game elements is needed, given the budget constraints. The narrative could have been presented without having to produce a 3D environment with animations and navigation. Immersion in digital games is not dependent on 3D representations but *is* affected by other factors, such as music and game mechanics.^45^ The writing of scenarios could have been emphasized instead, and professional game writers, as well as voice actors, could have been used to create a more immersive narrative experience.

Some of the participants expressed the desire for the inclusion of more complicated birth scenarios in the story-driven game in order to enhance their experience and permit them to engage in a wider range of scenarios, all of which could occur during or following childbirth. Also, the participants wanted the opportunity to further influence the scenarios. This reflects an interesting conflicting goal that was identified during development but was not resolved. The major goal of the *Childbirth Journey* is to put the players in the role of a parent so they can make the same choices parents must make. Many of the factors that affect the birth process are not, however, completely in the hands of parents. On the contrary, some decisions are made solely by professionals, whereas others are a matter of fate or faith. Some simulation games give players the power to “act as God” and to experiment with different parameters and scenarios. It is, however, highly problematic, from a user experience perspective, to have players moving between roles. The distinction between a single player's experience from a play session and the set of potential outcomes of a game was also an issue that was not clear to all healthcare professionals during development. If players are not primarily focused on exploring the set of potential outcomes (such as birth outcomes), it may not be necessary to create a large set of such outcomes (such as normal birth, caesarean section, or vacuum extraction delivery), particularly if they do not originate from decisions made by the player. One conclusion of this study in this respect is that healthcare professionals who seek to develop games must decide on the perspective assumed by the player. Also, it is important to reflect upon the consequences of providing expecting parents, or patients, the ability to experiment and explore different parameters, especially considering that some of these are not under their—or anyone's—control in reality. The present study indicates that the most important choice provided to players is the ability to choose a role and to engage in dialogue with their partners after using the *Childbirth Journey*.

In this study, the participants perceived the films and web links included in the story-driven game as a positive feature—in fact, these were the most appealing elements of the *Childbirth Journey*. However, they expressed the need for more films illustrating different types of pain relief, examinations, or various methods to handle pain due to labor contractions. Besides, they desired a larger variety of healthcare professionals to take part in the films. To conclude, it can be argued that this intervention did not satisfactorily meet the *detailed* or *entertaining* parts of the characteristics previously described as important for expecting mothers using digital media during pregnancy.^6^ All of the participants tested the mobile version and the Swedish version of the *Childbirth Journey*. The fact that all participants had knowledge of the Swedish language and, therefore, used the Swedish version of the *Childbirth Journey* may be seen as a limitation; hence, further exploration of the English version of the intervention is needed to explore cultural aspects. In future research, parents representing LGBTQ relationships should also be included to deepen knowledge concerning gender-related issues. Additionally, all participants had a university education, which could also be seen as a limitation since the educational level of expecting parents is associated with their ability to choose between conflicting information online.^46,47^ Further lessons learned from this intervention study is that interview questions on participants’ previous experiences using serious games experiences should be included as well as questions concerning more specific aspects of their user behavior with regard to the intervention (such as how much time they spent on the intervention and which parts of the game they had used). In future explorations of user experiences of digital parental support, methods that permit researchers to observe when and how parents use such support would be valuable. In this respect, a “think aloud research” approach could be appropriate. The strengths of this study, on the other hand, are that, in the study protocol for the larger research project, *Digital parental support,*^19^ it was assumed that the intervention could be experienced by the participants as time consuming. On the contrary, the participants perceived the intervention to be *time saving* instead.

In sum, despite the relatively limited number of parents interviewed, it is reasonable to conclude that the results of this pilot intervention study highlight both the strengths and limitations of the *Childbirth Journey* intervention as well as the need for improvement in several areas. In addition*,* it can be argued that the intervention could work as a valuable complement to regular professional support offered to expecting parents. In turn, expecting parents’ concerns and anxiety might be reduced by gaining enhanced, reliable and accurate knowledge and by obtaining a better understanding of childbirth and its preparation as a consequence of using the intervention. Because the health literacy of expecting parents influences their understanding about how to use the Internet for health-related information,^48^ low health literacy is associated with poorer health-related knowledge and a decreased capacity to correctly interpret health messages and use healthcare services.^49^ Besides, the results of this study could serve as a guide for healthcare professionals and researchers in the future development of digital parental support tools for parents, as presented in Supplemental Table 2. However, when developing digital tools and conducting research projects like this in the future, financial considerations should be addressed. More extensive financial support is needed not just for developing digital tools but also for routinely revising their content since obstetric care is continually evolving.

## Conclusion

It is clear that expecting parents need reliable and accurate information and preparation for childbirth and parenthood. Expecting parents typically obtain such information from both professionals and digital sources, such as the Internet. However, information obtained online can cause concern among expecting parents with regard to its accuracy and reliability. In contemporary society, human beings are dealing not just with the rapid development of digital sources but also with crises such as the current COVID-19 pandemic. The results of this study revealed that expecting parents perceive the *Childbirth Journey* intervention to be a valuable complement to professional support, which has some deficiencies, especially at present due to the current pandemic restrictions in Sweden (i.e. partners are not allowed to participate in routine meetings with midwives at the antenatal clinic, or, sometimes, at the labor ward during, and after, birth). Therefore, it is reasonable to conclude that it would be valuable to further develop and explore the *Childbirth Journey* intervention and, in a longer perspective, to implement the tool as a regular form of digital parental support, one which allows expecting parents to more easily access reliable and accurate health information as controlled and administered by healthcare professionals. However, further research on healthcare professionals’ perceptions of the intervention is also needed in order to broaden the knowledge base concerning the *Childbirth Journey* as an intervention for expecting parents.

## Supplemental Material

sj-docx-1-dhj-10.1177_20552076221097776 - Supplemental material for Expecting parents’ perceptions of the digital parental support “childbirth journey” constructed as a serious game—an intervention study Click here for additional data file. Supplemental material, sj-docx-1-dhj-10.1177_20552076221097776 for Expecting parents’ perceptions of the digital parental support “childbirth journey” constructed as a serious game—an intervention study by Caroline Bäckström, Tanja Rolfson, Henrik Engström, Rajna Knez and Margaretha Larsson in Digital Health

## References

[1] Guendelman S Broderick A Mlo H , et al. Listening to communities: Mixed-method study of the engagement of disadvantaged mothers and pregnant women with digital health technologies. J Med Internet Res 2017; 19: e240. 28679489 10.2196/jmir.7736 PMC5517821

[2] Fleming SE Vandermause R Shaw M . First-time mothers preparing for birthing in an electronic world: Internet and mobile phone technology. J Reprod Infant Psychol 2014; 32: 240–253.

[3] Oscarsson MG Medin E Holmström I , et al. Using the internet as source of information during pregnancy - a descriptive cross-sectional study among fathers-to-be in Sweden. Midwifery 2018; 62: 146–150. 29684793 10.1016/j.midw.2018.04.008

[4] Reis J Amorim M Melao N , et al. Digital transformation: a literature review and guidelines for future research. In: Rocha A Adeli H PReis LP Sostanzo S (eds) Trends and advances in information systems and technologies. WorldCIST’18. Switzerland: Springer, 2018, pp. 411–421.

[5] Lima-Pereira P Bermúdez-Tamayo C Jasienska G . Use of the internet as a source of health information amongst participants of antenatal classes. J Clin Nurs 2012; 21: 322–330. 22093043 10.1111/j.1365-2702.2011.03910.x

[6] Lupton D . The use and value of digital media for information about pregnancy and early motherhood: A focus group study. BMC Pregnancy Childbirth 2016; 16: 1–10. 27435182 10.1186/s12884-016-0971-3 PMC4950377

[7] Åsenhed L Kilstam J Alehagen S , et al. Becoming a father is an emotional roller coaster – an analysis of first-time fathers’ blogs. J Clin Nurs 2014; 23: 1309–1317. 23815546 10.1111/jocn.12355

[8] Da Costa D Zelkowithz P Letourneau N , et al. Healthydads.ca: What do men want in a website designed to promote emotional wellness and healthy behaviors during the transition to parenthood? J Med Internet Res 2017; 19: 1–1. 10.2196/jmir.7415 PMC5658653 29021126

[9] Andalibi N Lacombe-Duncan A Roosevelt L , et al. LGBTQ Persons’ use of online spaces to navigate conception, pregnancy, and pregnancy loss: an intersectional approach. ACM Trans. Comput.-Hum. Interact 2022; 29: 1–46.

[10] Donelle L Hall J Hiebert B , et al. Investigation of digital technology use in the transition to parenting: Qualitative study. JMIR Pediatrics Parenting 2021; 4: e25388. 33595440 10.2196/25388 PMC8078692

[11] Vickery M van Teijlingen E Hundley V , et al. Midwives’ views towards women using mHealth and eHealth to self-monitor their pregnancy: A systematic review of the literature. European Journal of Midwifery 2020; 4: 1–11. 33537637 10.18332/ejm/126625 PMC7839093

[12] Bäckström C Carlén K Larsson V , et al. Expecting parents’ use of digital sources in preparation for parenthood in a digitalised society - a systematic review. Digit Health 2022; 8: 1–15. 10.1177/20552076221090335 PMC9016606 35449713

[13] Barimani M Vikström A Rosander M , et al. Facilitating and inhibiting factors in transition to parenthood - ways in which health professionals can support parents. Scand J Caring Sci 2017; 31: 537–546. 10.1111/scs.12367 28144992

[14] Cowan CP Cowan PA . When partner become parents. The big life change for couples. Mahwah, NJ, USA: Lawrence Erlbaum Associates, 2000.

[15] Ekström-Bergström A Thorstensson S Bäckström C . The concept, importance and values of support during childbearing and breastfeeding - A discourse paper. Nurs Open 2022; 9: 156–167. 34741500 10.1002/nop2.1108 PMC8685869

[16] Banke G Berglund A Collberg P , et al. Antental care sexual and reproductive health. (national guidelines). Stockholm: SFOG, 2008.

[17] Musiimenta A Tumuhimbise W Pinkwart N , et al. A mobile phone-based multimedia intervention to support maternal health is acceptable and feasible among illiterate pregnant women in Uganda: Qualitative findings from a pilot randomized controlled trial. Digit Health 2021; 7: 2055207620986296. 33717497 10.1177/2055207620986296 PMC7917428

[18] Naul EL M . Why story matters: A review of narrative in serious games. J Educ Comput Res 2019; 58: 687–707.

[19] Bäckström C Engström H Knez R , et al. Digital tools as parental support – A study protocol describing prospective development and exploration of two digital tools for parents. Front Digit Health 2021; 3: 698969. 10.3389/fdgth.2021.698969 PMC8651613 34901924

[20] Stokes B . Video games have changed: time to consider, serious games. Develop Educ J 2005; 11.

[21] Marton F . Phenomenography: A research approach to investigating different understandings of reality. J Thought 1986; 21: 28–49.

[22] Caroline B Sandi C Shazima T , et al. Parents’ perceptions about future digital parental support-A phenomenographic interview study. Front Digit Health 2021; 3: 729697. 34778868 10.3389/fdgth.2021.729697 PMC8578718

[23] Marsh T . Serious games continuum: Between games for purpose and experiential environments for purpose. Entertain Comput 2011; 2: 61–68.

[24] Sjöström B Dahlgren L-O . Applying phenomenography in nursing research. J Adv Nurs 2002; 40: 339–345. 12383185 10.1046/j.1365-2648.2002.02375.x

[25] Sayakhot P Carolan-Olah M . Internet use by pregnant women seeking pregnancy-related information: A systematic review. BMC Pregnancy Childbirth 2016; 16: 65. 10.1186/s12884-016-0856-5 PMC4810511 27021727

[26] Lupton D Pedersen S . An Australian survey of women's Use of pregnancy and parenting apps. Women Birth: J Australian College Midwives 2016; 29: 368–375. 10.1016/j.wombi.2016.01.008 26874938

[27] Bjelke M Martinsson A-K Lendahls L , et al. Using the internet as a source of information during pregnancy – a descriptive cross-sectional study in Sweden. Midwifery 2016; 40: 189–191. 10.1016/j.midw.2016.06.020 27450590

[28] Schumacher K Meleis A . Transitions. A central concept in nursing. J Nurs Scholarsh 1994; 26: 119–127. 10.1111/j.1547-5069.1994.tb00929.x 8063317

[29] Meleis AI Sawyer LM Im EO , et al. Experiencing transitions: An emerging middle-range theory. Adv Nurs Sci 2000; 23: 12–28. 10.1097/00012272-200009000-00006 10970036

[30] Hildingsson I Haines H Johansson M , et al. Childbirth fear in Swedish fathers is associated with parental stress as well as poor physical and mental health. Midwifery 2014; 30: 248–254. 24445076 10.1016/j.midw.2013.12.012

[31] Peahl AF Smith RD Moniz MH . Prenatal care redesign: creating flexible maternity care models through virtual care. Am J Obstet Gynecol 2020; 223: 389. e1–389.e10. 32425200 10.1016/j.ajog.2020.05.029 PMC7231494

[32] AlJaberi H . Developing culturally sensitive mHealth Apps for Caribbean immigrant women to use during pregnancy: focus group study. JMIR Human Factors 2018; 5: e29–e29. 30305256 10.2196/humanfactors.9787 PMC6231776

[33] Song FW West JE lundy L , et al. Women, pregnancy, and health information online: the making of informed patients and ideal mothers. Gender Society 2012; 26: 773–798.

[34] Wells M . Literature review shows that fathers are still not receiving the support they want and need from Swedish child health professionals. Acta Paediatr 2016; 105: 1014–1023. 27310679 10.1111/apa.13501

[35] Wells M Lang S . Supporting same-sex mothers in the Nordic child health field: A systematic literature review and meta-synthesis of the ost gender equal countries. J Clin Nurs 2016; 25: 3469–3483. 10.1111/jocn.13340 27451972

[36] Pålsson P Persson EK Ekelin M , et al. First-time fathers experiences of their prenatal preparation in relation to challenges met in the early parenthood period: Implications for early parenthood preparation. Midwifery 2017; 50: 86–92. 28399472 10.1016/j.midw.2017.03.021

[37] Feinberg ME . Coparenting and the transition to parenthood: a framework for prevention. Clin Child Fam Psych 2002; 5: 173–195. 10.1023/a:1019695015110 PMC3161510 12240706

[38] Le Y McDaniel BT Leavitt CE , et al. Longitudinal associations between relationship quality and coparenting across the transition to parenthood: A dyadic perspective. J Fam Psychol 2016; 30: 918–926. 27183188 10.1037/fam0000217 PMC5112151

[39] Fivaz-Depeursinge E Favez N . Exploring triangulation in infancy: two contrasted cases. Fam Process 2006; 45: 3–18. 16615250 10.1111/j.1545-5300.2006.00077.x

[40] Ruggiero D Watson WR . Engagement through praxis in educational game design: common threads. Simul Gaming 2014; 45: 471–490.

[41] Passarellia M Earp J Dagnino FM , et al. The distant horizon: investigating the relationship between social sciences academic research and game development. Entertain Comput 2020; 34: 1–8.

[42] Holvikivi J Juurola L Nuorteva M . Collaboration platform for public and private actors in educational games development. Open Conf Comput Educ 2018: 141–150.

[43] Orji R Nacke LE Di Marco C . Towards personality-driven persuasive health games and gamified systems. Proceedings of the 2017 CHI Conference on Human Factors in Computing Systems 2017:1015–1027.

[44] Göbel S Hugo O Kickmeier-Rust M , et al. Serious games—economic and legal issues. In: *Serious Games*. Edited by Dörner R, Göbel S, Effelsberg W, Wiemeyer J. Cham: Springer.

[45] Cairns P Cox A Nordin AI . Immersion in digital games: review of gaming experience research. Handbook Digit Games 2014; 1: 337–361.

[46] Hämeen-Anttila K Nordeng H Kokki E , et al. Multiple information sources and consequences of conflicting information about medicine use during pregnancy: A multinational internet-based survey. J Med Internet Res 2014; 16: e60. 24565696 10.2196/jmir.2939 PMC3961698

[47] Vamos CA Merrell L Detman L , et al. Exploring Women's Experiences in accessing, understanding, appraising, and applying health information during pregnancy. J Midwifery Women's Health 2019; 64: 472–480. 31050386 10.1111/jmwh.12965

[48] Shieh C Mays R McDaniel A , et al. Health literacy and its association with the use of information sources and with barriers to information seeking in clinic-based pregnant women. Health Care Women Int 2009; 30: 971–988. 19809901 10.1080/07399330903052152

[49] Berkman ND Sheridan SL Donahue KE , et al. Low health literacy and health outcomes: An updated systematic review. Ann Intern Med 2011; 155: 97–107. 21768583 10.7326/0003-4819-155-2-201107190-00005

