# Supplementary material for: Expecting parents’ perceptions of the digital parental support “childbirth journey” constructed as a serious game—an intervention study
Source: Digit Health. 2022 May 16;8:20552076221097776. doi: 10.1177/20552076221097776 (PMC9118415; doi:10.1177/20552076221097776)
Supplement: sj-docx-1-dhj-10.1177_20552076221097776 - Supplemental material for Expecting parents’ perceptions of the digital parental support “childbirth journey” constructed as a serious game—an intervention study [file sj-docx-1-dhj-10.1177_20552076221097776.docx]

| **Table 2.** Descriptive categories and parents’ suggestions for improvement, based on experiences from the *Childbirth Journey* intervention | |
| --- | --- |
| **Descriptive categories** | **Parents’ suggestions** |
| Easily accessible, customised and reliable information | More scenarios, i.e. postnatal care at the hospital, first time at home with the child  Access to scenarios not yet developed in the present version  More practical information about preparation at home and what to take to the hospital  More scenarios describing complicated childbirth, such as a caesarean section or a post-partum haemorrhage  More films structured as constructive and information-rich lectures  More films on breathing techniques, massage techniques, other types of pain relief, complicated birth, breastfeeding, and the first time at home with the baby  Include various midwives, or other healthcare professionals, in contributing to the production of the films |
| Design and features influence usability, interest and trustworthiness | Synthetic speech should be replaced with human voice recordings, as a human narrator’s voice could emphasise different expressions  Include a greater variety of sounds in the story-driven game  Graphics in the story-driven game could be improved, e.g. animations need improvements to become more trustworthy  A return function that allows the user to go back in the text  Clickable links in the story-driven game  Story-driven game needs to be sped up |
| The parents’ mutual preparation creates an opportunity for conversation and facilitates understanding | - |
